# The association between subjective anti-doping knowledge and objective knowledge among Japanese university athletes: a cross-sectional study

**DOI:** 10.3389/fspor.2023.1210390

**Published:** 2023-11-16

**Authors:** Yuka Murofushi, Etsuko Kamihigashi, Yujiro Kawata, Shinji Yamaguchi, Miyuki Nakamura, Hanako Fukamachi, Hiroshi Aono, Yuji Takazawa, Hisashi Naito

**Affiliations:** ^1^ Faculty of Health and Sports Science, Juntendo University, Inzai-shi, Japan; ^2^ Graduate School of Health and Sports Science, Juntendo University, Inzai-shi, Japan; ^3^ Japan High Performance Sport Center, Tokyo, Japan; ^4^ Institute of Health and Sports Science and Medicine, Juntendo University, Inzai-shi, Japan; ^5^ Division of Public Health, Department of Hygiene and Public Health, School of Medicine, Tokyo Women's Medical University, Tokyo, Japan; ^6^ Japan Sport Association, Tokyo, Japan; ^7^ Graduate School of Medicine, Juntendo University, Tokyo, Japan; ^8^ Faculty of Medicine, Juntendo University, Tokyo, Japan

**Keywords:** theory of planned behavior, world anti-doping code, anti-doping education, anti-doping knowledge, objective knowledge, subjective knowledge

## Abstract

**Introduction:**

This study aimed to assess the association between *subjective anti-doping knowledge* (*subjective ADK*) and *objective anti-doping knowledge* (*objective ADK*) among Japanese university athletes, framed within the context of the *Theory of Planned Behavior* (TPB).

**Methods:**

Eligible participants were 486 university athletes [320 men (65.8%), 166 women; mean age of 18.9 ± 1.0 years]. The participants categorized themselves in terms of the quality of their *anti-doping* knowledge. This assessment resulted in an independent variable coded as “(1) substantial lack of adequate knowledge,” “(2) some lack of adequate knowledge,” “(3) fair amount of knowledge” or “(4) good amount of knowledge.” Objective *ADK* was assessed using the Athlete Learning Program about Health and Anti-Doping (ALPHA) test, a set of questions derived from the ALPHA—a former World Anti-Doping Agency e-learning program. The test comprises 12 questions (four choices each; passing index: ≧10 points or 80% correct answer rate). ANCOVA was conducted using *subjective ADK* as an independent variable and ALPHA scores as a dependent variable, adjusting for confounding factors (anti-doping experience).

**Results:**

The ALPHA corrected answer rate across *subjective ADK* levels for the group were 73.10% for “(1) substantial lack of adequate knowledge,” 71.97% for “(2) some lack of adequate knowledge,” 75.18% for “(3) fair amount of knowledge” and 72.86% for “(4) good amount of knowledge.” Comparison between different levels of *subjective ADK* revealed no significant differences in ALPHA score considering the main effects or any of their interactions.

**Discussion:**

The present results revealed that Japanese university athletes’ *subjective ADK* did not match their *objective ADK*. In the context of the *TPB*, there may be limitations in the perceived behavioral control in anti-doping knowledge. Even if athletes view doping as a wrongful act and have formed attitudes and subjective norms to comply with the rules, the results suggest that errors may occur in the composition of behavioral intentions due to a lack of knowledge. This could lead to the possibility of facing the risk of unintentional anti-doping rule violations. It highlights the need for targeted educational interventions to align subjective *ADK* of athletes with their *objective ADK*.

## 1. Introduction

Maintaining the integrity and fairness of competitive sports hinges on effective anti-doping efforts. Education is expected to prevent unintentional anti-doping rule violations (ADRVs). However, previous studies have revealed deficiencies in both athletes’ *subjective anti-doping knowledge* (*subjective ADK*) (1, 2) and their *objective anti-doping* knowledge (*objective ADK*) (3–5). This underscores the possibility of an unexplored gap or misalignment between the perceived and actual knowledge of an athlete. Tversky and Kahneman (6) discussed that empirical heuristic judgments can sometimes be accurate, but individuals might overestimate their abilities, potentially leading to mistaken choices (7). Athletes especially those exposed to anti-doping education or having doping control experience might fall prey to overconfidence. This phenomenon is exemplified by the Dunning–Kruger effect (8), where individuals with limited knowledge misjudge their actual expertise. Such cognitive biases, rooted in assumptions or misconceptions, can mislead decisions and potentially lead athletes with perceived sufficient anti-doping knowledge to make errors in judgment.

Along with anti-doping efforts, strategies for illicit performance enhancement continue to evolve. This ever-evolving landscape demands heightened vigilance, particularly for younger athletes who are notably vulnerable due to their impressionability (9, 10). Alongside these concerns, ethical debates surrounding the use of performance-enhancing drugs further complicate the narrative. The prevalence and perception of these drugs challenge the ethos of sports and fairness, have health implications, and are influenced by societal pressures (11–13). Emphasizing the dynamic nature of performance-enhancing drugs ensuring continuous and adaptive anti-doping education targeting both seasoned athletes and the youth is crucial. Despite these broader ethical and societal challenges, there are specific pitfalls within athletes’ understanding and perceptions that directly affect their behaviors and decisions related to doping. Such gaps can be perilous, potentially leading to unintentional ADRVs. To better understand and address these challenges, exploring theoretical frameworks that shed light on athletes’ behaviors and decision-making processes is essential.

One such framework is the *Theory of Planned Behavior* (*TPB*), which provides a foundational framework for assessing doping risks. Rooted in the idea that attitudes, subjective norms, and perceived behavioral control guide the actions of an individual, *TPB* underscores the nuances of athletes’ choices (14). For instance, while athletes might intend to avoid doping, limited knowledge could constrain their behavior (15). The key variables of *TPB*, especially doping attitude and perceived behavioral control, have been linked to doping behaviors and intentions (16–18). Thus, enlightening athletes on their knowledge limitations becomes crucial. Understanding doping definitions and rules, as defined by the World Anti-Doping Agency (WADA) Code, is also essential. Doping is defined as the occurrence of one or more of the 11 ADRVs outlined in the code (19). It includes not only the use of prohibited substances or methods but also possession, enabling others’ use or non-disclosure of whereabouts. Also, the code differentiates between intentional and unintentional doping. Recognizing this, a robust strategy is needed to prevent both types of ADRV effectively. Code signatories, including National Anti-Doping Organizations and each athletic organization that is a signatory to the code, have been mandated to promote anti-doping education since the formulation of the 2021 Code International Standard for Education (ISE) (19, 20). Given this, a more effective preventive approach to both intentional and unintentional ADRV is required.

Chan et al. (21) suggested that a lack of anti-doping knowledge can lead to unintentional ADRVs. Ntoumanis et al. (22) suggest that anti-doping knowledge may lead to more adaptive responses to doping-related cognitions and behaviors. Thus, improving anti-doping knowledge should contribute to one aspect of the prevention of unintentional ADRVs. However, studies focusing on countries or sports with high ADRVs have included some negative opinions about anti-doping education. A few studies suggest that the knowledge and information provided may not prevent doping (23–27). Based on the above, rather than solely focusing on improving anti-doping knowledge as an educational strategy for doping prevention, a comprehensive approach that includes improving practical knowledge is desirable.

However, anti-doping education previously primarily targeted elite athletes and focused on prohibited substances and methods or doping control and sanctions (25). Nevertheless, it has been observed that even top-level athletes often lack practical anti-doping knowledge (4). Against this background, in numerous countries, anti-doping education has been conducted for youth athletes, who are expected to compete at the international level in the future (26, 53), and for university-age athletes, including recreational athletes (3, 4, 29–31). Unfortunately, most studies pointed to athletes’ lack of anti-doping knowledge among athletes, especially in terms of medical knowledge such as the side effects of doping (1, 2, 4, 32), and discrepancies between *subjective* and *objective ADK* could potentially increase doping risk. Moreover, discrepancies between subjective and objective ADK could distort these attitudes, leading to potentially risky doping decisions. Notably, a meta-analysis by Ntoumanis et al. (22) found that attitudes, perceived norms, and self-efficacy to refrain from doping were significant predictors of doping intentions and behaviors.

Despite limited studies examining athletes’ perceptions of doping risk or their *subjective ADK* (33), it is clear from human cognition research that discrepancies between *subjective* and *objective ADK* can lead to risky behavior. For example, some methods measure the amount of knowledge based on subjective reports and evaluations of survey participants (34, 35). Other methods use a quiz format and measure the amount of scientific knowledge based on the number and percentage of correct answers, such as by judging correct or incorrect answers (36, 37). However, no research has yet focused on the relationship between *subjective* and *objective ADK* in the anti-doping field. Relying solely on subjective knowledge does not lead to appropriate decisions or behavioral choices, and in particular, the risk of unintentional ADRV pitfalls is foreseeable.

This study aimed to investigate the impact of *subjective ADK* on *objective ADK* among Japanese university athletes within the *TPB* framework. Clarifying these issues is expected to contribute to the development of a more effective anti-doping education program.

## 2. Materials and methods

### 2.1. Participants

This study used a cross-sectional design. The eligible participants were 486 Japanese university athletes [320 (66%) men, 166 (34%) women; mean age = 18.9 ± 1.0 years] affiliated with Japanese sports universities. Given that the ratio of men to women university athletes in Japan is approximately 6:4 to 7:3, the participant population of this study is considered to reflect a general population ratio. The criteria for athletes in this study were defined as being engaged in sports activities to participate in competitions. All individual competition levels were included in the survey. A total of 538 individuals were initially approached, taking into account potential missing data or dropouts. Out of these, 508 met the inclusion criteria, whereas 22 were excluded for not meeting the athlete criteria.

To ensure adequate statistical power for the analyses, we conducted a post-hoc power analysis using the G*Power software (38). The parameters were set for a one-way ANOVA with an effect size *f* of 0.15, *α*-level of 0.05, and power of 0.80 across four groups. The analysis suggested a required sample size of 492 to reach the desired power level. The achieved sample size of 486 was very close to this estimate, resulting in an actual power of 0.803.

### 2.2. Survey items

#### 2.2.1. Demographic data of participants

The demographic data of the participants included sex, age, sports event, individual competition level [recreational athletes (district and prefecture), national, and international], and years of competition experience (≦5, 6–10, 11–15, ≧16 years). The “individual competition level” refers to the highest level of competition in which an athlete has personally participated. For the purpose of this study, this level was categorized into recreational athletes (district and prefecture-level), national-level athletes, and international-level athletes. We also asked for experience in anti-doping education and doping control experience (experienced, non-experienced). There is no consistent anti-doping education in Japan, hence we asked respondents about self-reported anti-doping education frequency.

#### 2.2.2. Measurement of subjective anti-doping knowledge

We asked athletes to rate their current *subjective ADK* status with the statement, “I have adequate knowledge about anti-doping.” The participants could select one of the following four options: “(1) substantial lack of adequate knowledge,” “(2) some lack of adequate knowledge,” “(3) fair amount of knowledge,” and “(4) good amount of knowledge” (score range: 1–4). All provided response was positioned as a level of *subjective ADK*.

#### 2.2.3. Measurement of objective anti-doping knowledge [Athlete Learning Program about Health and Anti-Doping (ALPHA) test]

At present, there is no uniform survey method to measure *objective ADK*. Therefore, the *objective ADK* assessment test used in the studies by Murofushi et al. (4) was adopted. This questionnaire was utilized for the knowledge assessment test in the e-learning program content of WADA, known as ALPHA until 2020. The ALPHA program content includes a knowledge confirmation test at the beginning and end of the e-learning program (39). Within this paper, the said test will be referred to as the ALPHA test for brevity. The ALPHA test consists of 12 questions, each of which was answered by selecting one of four options. After the ALPHA test, a certificate was issued if the correct answer rate was 80% or more (e.g., a score of 9.6 or more when converting to points). For each of the 12 correct answers, the final evaluation score was the sum of the numerical values, which were 1 for correct answers and 0 for incorrect answers (score range: 0–12 points). The correct answer rate was also calculated from the total score. This study used a score of 10 points (80% or higher), an indicator for passing the ALPHA test, as the evaluation index. The pass index for each ALPHA question was defined as 80%, which is the total score index of the ALPHA test. As reference information, the choice rates for the selected branches for each ALPHA question, the index of discrimination (D score) (40), and the results of the I-T correlation analysis are shown in Supplementary Tables 1–3. Although some items did not have sufficient discriminant indices, all item scores were included in the analysis to allow for comparison with previous studies.

### 2.3. Survey period and procedure, ethics statement

We used Google Forms to survey athletes from three Kanto region universities integrated anti-doping curricula between May and December 2020. The survey was conducted online, and the accessibility of the questionnaire was taken into account, which led to extensive data collection and rapid collection and the prevention of misstatements and errors in data entry that are likely to occur in paper-based data collection. Although the online nature of the survey could theoretically introduce a risk of inauthentic responses, it was assumed that the differences between the online and paper questionnaires would be negligible, especially as the questions were similar to established psychometric scales. No specific provision was made for the number of days between the completion of the course and the completion of the questionnaire, as the lecture schedule depended on the university curriculum. However, we requested that participants respond to the survey before the lecture was given and informed them verbally and on the web page that the survey would be completed 1 week after the date of the request. The researcher explained the purpose of the survey, and those who provided informed consent were asked to complete the survey during the lecture hours of each university or self-study hours of each student. The responses to the questionnaire were collected anonymously.

The survey took approximately 10–15 min. The participants could stop answering anytime not later withdrawn. The study was reviewed and approved by the research society ethics committee of the Faculty of Health and Sports Science and the Graduate School of Health and Sports Science, Juntendo University, Japan (No. 2022–74). The participants provided their written informed consent to participate in this study. The aims of the research were fully explained to participants, and the survey data were collected only when informed consent was obtained. We also demonstrated that privacy would not be violated and that data exclusion would be almost impossible after collection. In the unlikely event that any mishap, such as mental distress, occurred during the survey response, the participants were informed that they could withdraw their consent to the study and discontinue their participation at any time, without any penalty.

### 2.4. Analysis method

#### 2.4.1. Calculation of demographic data, subjective anti-doping knowledge, and ALPHA scores of participants

Descriptive statistics were applied to evaluate the demographic data, doping control experience, and anti-doping education of the participants aiming to calculate both the number and proportion (percentage) of participants in each category. Subsequently, we determined the mean and standard deviation (SD) for the *subjective ADK* and ALPHA scores, based on the demographic data. Using cross-tabulation, we calculated the percentage of respondents who chose “(3) fair amount of knowledge” and “(4) good amount of knowledge” for the subjective ADK question, segmented by demographic data. Lastly, the ALPHA correct answer rates were tabulated based on the demographic data.

#### 2.4.2. Comparison of ALPHA scores by subjective anti-doping knowledge

We identified the confounding factors that influenced the *subjective ADK* and ALPHA scores. A MANOVA was conducted using demographic data, doping control experience, and anti-doping education experience as independent variables and the *subjective ADK* and ALPHA scores as dependent variables.

Subsequently, we calculated the number and percentage of *subjective ADK* levels using descriptive statistics.

As the principal analysis of this study, the ALPHA scores with the *subjective ADK* levels [(1) *substantial lack of adequate knowledge* to (4) *good amount of knowledge*] were compared. An ANCOVA was conducted with the *subjective ADK* levels set as the independent variable and ALPHA scores as the dependent variable, adjusting for confounding factors. Bonferroni correction was applied for subsequent multiple comparisons.

#### 2.4.3. Comparison of correct answer rate per ALPHA question by the level of subjective anti-doping knowledge

First, for each of the 12 ALPHA questions, the ALPHA correct answer rate by the level of *subjective ADK* was calculated. Next, MANOVA was conducted to identify confounding factors affecting the correct answer rate of each ALPHA question and *subjective ADK*. The demographic data, doping control experience, and anti-doping education experience of the participants are independent variables, and each ALPHA question's correct answer rates are dependent variables.

Subsequently, depending on the identification of confounding factors for each ALPHA question, either an ANCOVA or ANOVA was conducted. An ANCOVA was conducted by setting the level of *subjective ADK* as the independent variable, ALPHA score as the dependent variable, and confounding factors as the covariates. For questions without identified confounding factors, ANOVA was performed. For subsequent multiple comparisons, when a significant difference was found, the Bonferroni method was used to adjust the significance level for multiple comparisons.

#### 2.4.4. Effect size, significance level, and statistical analysis tools

For the comparison of ALPHA scores by *subjective ADK* analysis and correct answer rate by the level of *subjective ADK* for each ALPHA question, we calculated the effect size. This was then assessed based on Cohen's criterion of relevance (small = 0.01, medium = 0.06, large = 014.) (41, 42). The significance level for all analyses was set at ≤5%. All statistical analyses were conducted using the statistical analysis software IBM SPSS Statistics Advanced 28 (IBM, Tokyo, Japan).

## 3. Results

### 3.1. Demographic data, subjective anti-doping knowledge, and ALPHA scores of participants

The demographic data of the participants are shown in Table 1. The individual competition level was highest for the national-level athletes, followed by recreational athletes who compete both at the prefecture and district level and finally the international-level athletes. The participants participated in various sports events. The most prevalent sports among participants were athletics (22.84%), football (15.02%), basketball (8.64%), baseball (8.64%), and volleyball (8.44%). Further details of the distribution across sports events are listed in Supplementary Table 4. The percentage of athletes with doping control experience was 4.73% (*n* = 23). Nearly 80% of the total athletes had anti-doping education.

**Table 1:** Demographic data of the participants.

Category		*n*	(*n*%)
Sex	Men	320	65.84
	Women	166	34.16
Athletic event (total of 33 sports)	Team sports (13 sports)	172	35.39
	Individual sports (20 sports)	304	62.55
	Other sports	10	2.06
Individual competition level	Recreational athlete (district)	41	8.44
	Recreational athlete (prefecture)	191	39.30
	National level athlete	228	46.91
	International level athlete	26	5.35
Competition duration (years)	<5 years	27	5.56
	6–10 years	111	22.84
	11–15 years	235	48.35
	≧15years	113	23.25
Anti-doping education experience	Experienced	94	80.66
	Non-experienced	392	19.34
Doping control experience	Experienced	23	4.73
	Non-experienced	463	95.27

*n*, number of eligible participants; (*n*%), percentage of eligible participants. Individual sports: athletics, swimming, gymnastics, and others. Team sports: football, baseball, basketball, and others.

The *subjective ADK* scores (SD) by participant demographic data are listed in Table 2. In addition, ALPHA scores (SD) and correct answer rates are also provided in the same table.

**Table 2:** *Subjective anti-doping knowledge* and ALPHA score by demographic data.

Category	Classification	Subjective anti-doping knowledge score^a^			ALPHA score^c^		
		Mean	SD	% of answered knowledgeable^b^	Mean	SD	Correct answer rate (%)^d^
Sex	Men	2.56	0.75	54.38	8.67	2.24	72.27
	Women	2.53	0.71	56.02	9.16	1.86	76.31
Individual competition level	Recreational athlete (district)	2.46	0.71	48.78	9.29	1.78	77.44
	Recreational athlete (prefecture)	2.50	0.70	53.40	8.96	1.89	74.65
	National-level athlete	2.61	0.76	57.89	8.68	2.31	72.30
	International-level athlete	2.46	0.81	50.00	8.65	2.48	72.12
Years of competition experience (years)	≦5	2.44	0.80	55.56	7.85	2.14	65.43
	6–10	2.58	0.78	55.86	8.75	2.35	72.90
	11–15	2.63	0.71	60.43	9.08	1.94	75.64
	>15	2.39	0.70	42.48	8.66	2.19	72.20
Anti-doping education experience	Experienced***	2.63	0.70	58.93	8.91	2.06	74.25
	Non-experienced	2.22	0.79	38.30	8.55	2.38	71.25
Doping control experience	Experienced	2.96	0.48	86.96	8.65	2.72	72.10
	Non-experienced	2.53	0.74	53.35	8.85	2.10	73.72

SD, standard deviation.

^a^ Questioned “I have adequate knowledge about anti-doping,” and selected one from (1) *substantial lack of adequate knowledge*, (2) *some lack of adequate knowledge*, (3) *fair amount of knowledge*, and (4) *good amount of knowledge* (score rage:1–4 points).

^b^ % of answered knowledgeable: percentage of participants who answered (3) *fair amount of knowledge*, and (4) *good amount of knowledge*.

^c^ 0–12 points, passing index of >80%.

^d^ Correct answer rate (%): correct answer rate for ALPHA scores.

*** *p* < .001. “Anti-doping education experience” was identified as a confounding factor for *subjective ADK* scores via MANOVA.

The mean overall *subjective ADK* score was 2.55 ± .73 points. The athletes who answered “(3) fair amount of knowledge” for subjective ADK accounted for 47.73% (*n* = 232) and “(4) good amount of knowledge” accounted for 7.20% (*n* = 35); the percentage of participants who answered to knowledgeable options was 54.93%. Moreover, 58.93% of the participants in anti-doping education and 86.96% of those with doping control answered with either “(3) fair amount of knowledge” or “(4) good amount of knowledge” regarding their subjective *ADK*.

The overall mean ALPHA score was 8.84 ± 2.13 points, equating to a 73.7% correct answer rate, which is below the 80% as a passing index. None of the demographic data, doping control, or anti-doping education experience attributes reached the ALPHA passing index score (≧80%). With regard to the distribution of ALPHA scores across the participants in this study, 43.83% (*n* = 213) of the athletes scored a passing index.

### 3.2. ALPHA scores by subjective anti-doping knowledge

Using MANOVA, we first identified the confounding factors affecting the *subjective ADK* scores and ALPHA scores. Significant differences were found among anti-doping education experience and *subjective ADK*, with the strength of the relationship between the variables ranging from small to medium levels [*F* (1, 422) = 14.139, *p* = 0.001, *η*^2^ = 0.03, 95% *CI* (.288 –.764)] (Table 2). Based on these findings, the anti-doping education experience was identified to be a confounding factor. Next, there was no significant difference between any of the variables and the ALPHA scores, and little relationship was found between the variables.

Next, we calculated the ALPHA scores across *subjective ADK* levels for the group. The ALPHA score was 8.77 ± 2.20 points (correct answer rate: 73.10%) for “(1) substantial lack of adequate knowledge,” 8.64 ± 2.15 points (71.97%) for “(2) some lack of adequate knowledge,” 9.02 ± 2.06points (75.18%) for “(3) fair amount of knowledge,” and 8.74 ± 2.3 6points (72.86%) for “(4) good amount of knowledge.” The population distribution by *subjective ADK* level was as follows: (1) *Substantial lack of adequate knowledge* was selected by 35 respondents, (2) *some lack of adequate knowledge* was selected by 184 respondents, (3) *fair amount of knowledge* was selected by 232 respondents, and (4) *good amount of knowledge* was selected by 35 respondents.

**Figure 1:**
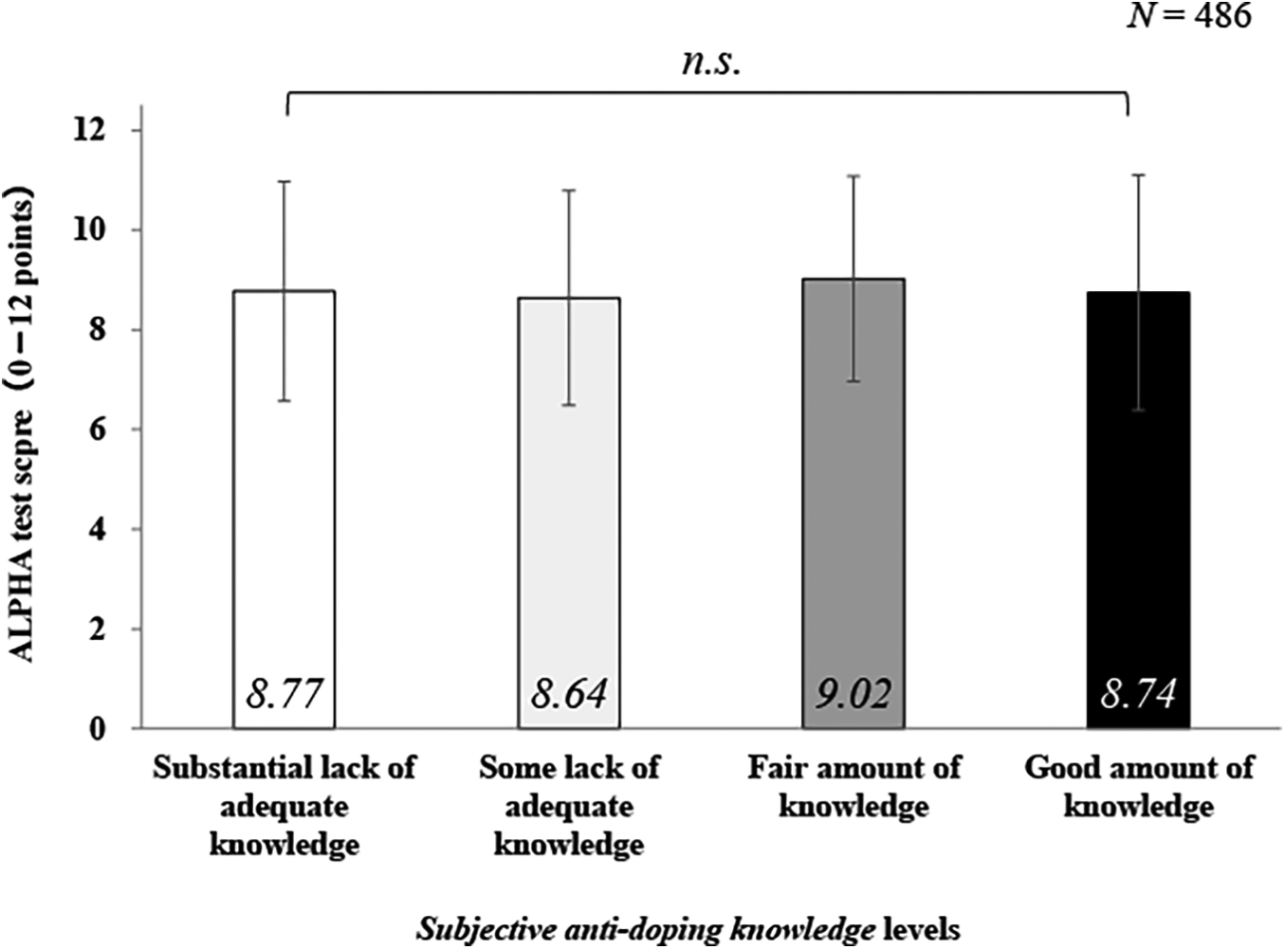
Comparison of subjective anti-doping knowledge and ALPHA scores. *Note*. n.s., not significant; *N*, number of participants. ALPHA score range: 0–12 points. ALPHA passing index of ≦80%. Subjective anti-doping knowledge score: questioned “I have adequate knowledge about anti-doping,” and selected one from (1) *substantial lack of adequate knowledge*, (2) *some lack of adequate knowledge*, (3) *fair amount of knowledge*, and (4) *good amount of knowledge* (score range: 1–4 points).

In our principal analysis, we conducted an ANCOVA using *subjective ADK* as the independent variable and ALPHA scores as the dependent variable and adjusted for anti-doping education experience as confounding factors. The results are shown in Figure 1. A two-factor analysis of variance between participants revealed no significant ALPHA score differences in the main effects of *subjective ADK* [*F* (3, 478) = 0.030, *p* = 0.993, *η*^2^ < 0.01], or interaction [*F* (3, 478) = 0.993, *p* = .963, *η*^2 ^< 0.01].

### 3.3. Correct answer rate per ALPHA question by the level of subjective anti-doping knowledge

To identify the confounding factors affecting each ALPHA question and *subjective ADK*, we noted significant differences, particularly between doping control experience and Q4 [*F* (1,422) = 4.434, *p* = .036, *η*^2^ = 0.01] individual competition level, Q6 [*F* (3,422) = 3.076, *p* = .027, *η*^2^ < 0.01], and Q8 [*F* (3,422) = 2.627, *p* = .050, *η*^2^ < 0.01].

An ANCOVA was conducted with the above confounding factors as covariates in the analysis of each ALPHA question. For other questions where no confounding factors were identified, an ANOVA was performed. The correct answer rates for each ALPHA question were compared by the level of *subjective ADK*. Significant differences were found for Q9 {[*F* (3,482) = 3.046, *p* = .028, *η*^2^ = 0.02]} and Q10 {[*F* (3,482) = 2.860, *p* = .037, *η*^2^ = 0.02]} (Table 3). Multiple comparisons showed no significant differences in either.

**Table 3:** Correct answer rate per ALPHA question by *subjective anti-doping knowledge* level.

no.	ALPHA question content	All *n* = 486 (%)	ALPHA correct answer rate (%) by *subjective anti-doping knowledge* level*s*				*p*	*η* ^2^
			Substantial lack of adequate knowledge	Some lack of adequate knowledge	Fair amount of knowledge	Good amount of knowledge		
1	What is the philosophy behind anti-doping?	87.04	85.71	88.04	87.07	82.86	0.860	<0.01
2	What is the purpose of the World Anti-Doping Code?	68.52	54.29	69.02	70.69	65.71	0.269	0.01
3	What is the prohibited list?	87.04	82.86	86.96	88.36	85.71	0.814	<0.01
4	What are the side effects of using anabolic steroids?	45.47	34.29	43.48	47.41	54.29	0.219	0.01
5	What does TUE stand for?	89.09	94.29	89.67	88.36	85.71	0.667	<0.01
6	How can an athlete with a medical condition decide whether to take a medication?	70.99	65.71	70.11	72.84	68.57	0.811	<0.01
7	Who is responsible for the substances found in an athlete's body?	95.47	94.29	93.48	96.98	97.14	0.355	0.01
8	What condition allows an athlete to refuse to be tested?	89.30	88.57	86.96	91.38	88.57	0.539	<0.01
9	When must an athlete be notified of an upcoming test?	68.72	54.29	66.85	74.14	57.14	0.028^†^	0.02
10	When do athletes have to tell their National Anti-Doping Organization where they will be living, training, and competing?	41.36	54.29	33.70	44.40	48.57	0.037^†^	0.02
11	What are the athlete's rights when a positive test is returned?	67.49	54.29	68.48	67.24	77.14	0.227	0.01
12	What is the requirement for laboratories that analyze blood or urine samples for doping control?	73.66	68.57	72.28	75.86	71.43	0.727	<0.01

ALL *n*, number of eligible participants. ALPHA correct answer rate (%): percentage of correct answers per ALPHA question (ALPHA passing index ≧80%). Subjective anti-doping knowledge score: questioned “I have adequate knowledge about anti-doping,” and selected one from (1) *substantial lack of adequate knowledge*, (2) *some lack of adequate knowledge*, (3) *fair amount of knowledge*, and (4) *good amount of knowledge* (score range: 1–4 points).

Bold and underlined: The ALPHA score with the highest correct answer rate for each question item is shown in bold and underlined.

^†^ Results of multiple comparisons showed no significant differences.

We then analyzed the proportion of items with the highest and lowest correct answer rates within the 12 ALPHA items. Below is the highlight of correct answer rates:

Highest rates: Q5 “What does TUE stand for?”, Q7 “Who is responsible for the substances found in an athlete's body?”, and Q8 “What condition allows an athlete to refuse to be tested?” stood out with rates between 85%–97% across all *subjective ADK* levels.

Lowest rates: Q4 “What are the side effects of using anabolic steroids?” and Q10 “When do athletes have to tell their National Anti-Doping Organization where they will be living, training, and competing?” had the lowest rates, especially at the “substantial lack of adequate knowledge” and “some lack of adequate knowledge” levels.

Noteworthy, at the “fair amount of knowledge” level, six items (Q2, Q3, Q6, Q8, Q9, and Q12) had the highest rates, while at the “good amount of knowledge” level, three items (Q4, Q7, Q11) stood out.

## 4. Discussion

### 4.1. The discrepancy between subjective and objective anti-doping knowledge

This study aimed to investigate the relationship between subjective and *objective ADK* in Japanese university athletes. Shedding light on this relationship could pave the way for the creation and enhancement of more efficient anti-doping education programs. We examined the effect of *subjective ADK* on ALPHA scores and found no significant differences. In other words, our findings reveal a discrepancy between athletes’ *subjective* and *objective ADK*. Approximately 55% of the athletes in this study perceived themselves as having “good amount of knowledge” regarding their *subjective ADK*. This is broken down into 47.73% who chose (*3) fair amount of knowledge* and 7.20% who selected *(4) good amount of knowledge*. Muwonge et al. (1) reported that less than 40% of professional-level athletes expressed confidence in their anti-doping knowledge, suggesting a majority lacked adequate understanding. Similarly, the level of *subjective ADK* regarding the definition of doping was found to be low among these athletes. Kim et al. (2) found that the subjective knowledge of prohibited substances was deemed inadequate by 39.0% of adolescent elite athletes and 53.4% of adult elite athletes. Consequently, the findings of our study align with these previous reports.

The influence of anti-doping education on *subjective ADK* perceptions is also evident. Athletes with anti-doping education experience tended to rate their *subjective ADK* higher than those without such experience. Cognitive biases, as described by Tversky and Kahneman (6) and exemplified by the Dunning–Kruger effect (7, 8), might explain this discrepancy. In addition, this gap between *subjective* and *objective ADK* could be seen as a disconnect between behavioral intention and actual behavior (14). It suggests that even if athletes believe they are well-informed, this does not necessarily translate into objectively measured knowledge or compliance with anti-doping rules. This disconnect between perceived and actual knowledge underscores the importance of considering subjective evaluations in anti-doping educational endeavors, especially given the inherent challenges in accurately gauging one's own knowledge depth (43).

### 4.2. Objective knowledge assessment insights from ALPHA scores

#### 4.2.1. Discrepancies in correct answer rate

ALPHA scores did not reach the passing index of ≥80% or 10 points. In particular, the correct answer rate for questions about anabolic steroid side effects (Q4) and whereabouts (Q10) was low. A previous study noted that Japanese university athletes had a low correct answer rate for the side effects of doping (Q4); the same was true for the participants in this study (4). In contrast, athletes with more experience in anti-doping education showed higher correct answer rates to these questions (44). A survey of *subjective ADK* among adolescent athletes found a certain level of understanding of the health hazards of prohibited substances but a low level of knowledge of actual side effects (45). In a self-reported survey of elite athletes, those who viewed prohibited substances as a minor health risk were more likely to use them than those who viewed them as a significant health risk (46). There is an emphasized need for education programs due to knowledge gaps and limited research on health risks among Japanese university athletes. Hence, it is necessary to investigate the situation and link it to appropriate educational interventions. Notably, although there is evidence that increased engagement with anti-doping education can lead to higher ALPHA scores (4) and positively affect the correct answer rates (44), many athletes, even those with extensive anti-doping education experiences, still fail to meet the passing threshold for the ALPHA test. This continues to underscore the knowledge deficit prevalent among university athletes.

#### 4.2.2. Areas of strength and weakness in anti-doping knowledge

The questions related to doping control (Q3), prohibited list (Q5), TUE (Q7), athletes’ responsibility (Q8), and refusal to doping control (Q9) consistently achieved an achieved an 80% correct answer rate for all *subjective ADK* levels. These results indicate that the respondents have basic knowledge in this education category. These highlighted the need for an educational approach to address a category of athletes with insufficient knowledge among the 11 anti-doping education topics recommended in the ISE (20). The history and current design of anti-doping educational programs have shown limited effectiveness, as evidenced by ongoing gaps in athlete knowledge (47). However, in some cases, as in the results of this study, athletes may perceive their anti-doping knowledge to be adequate. Therefore, it is crucial to discern whether athletes are potentially overlooking essential information.

### 4.3. Addressing knowledge gaps and the role of education and prevention

#### 4.3.1. *TPB*-guided assessment on doping risks and the need for targeted education

Although the number of ADRVs among Japanese athletes tended to be low, there is an increasing concern regarding violations attributed to supplement use for performance-enhancing effects and those due to medication taken for therapeutic purposes. The risk posed by supplements is multifaceted: contamination during the manufacturing processes of supplements (48) and the potential presence of undeclared doping substances (49, 50). A notable study spanning 18 years highlighted that 26% of all ADRV cases could be linked to dietary supplements, of which half were directly backed by evidence (51). Some of these supplements may be ineffective or even harmful, containing banned substances (52). This highlights the increased risks that athletes face when choosing supplements without a thorough understanding.

To mitigate the risks associated with this knowledge, a multi-faceted approach, including targeted surveys and tailored preventive education programs, could be effective. The *TPB* suggests that attitudes, subjective norms, and perceived behavioral control all contribute to the likelihood of engaging in a particular behavior. Therefore, a comprehensive educational program that addresses all of these components may be more effective in bridging the knowledge gap and reducing the risk of doping. Attitudes toward doping, subjective norms, and perceived behavioral control factors increase the likelihood that athletes will have the behavioral intention not to dope. The degree to which athletes are convinced that they can avoid doping on their own is important; for example, beliefs that athletes are confident in their abilities and not dependent on external factors such as doping and other psychological system variables influence and shape behavioral intentions to avoid doping. Given this background, it becomes imperative to identify the most effective strategies to convey this knowledge and guide athletes toward making informed decisions.

#### 4.3.2. Strategies for effective anti-doping education

Furthermore, our findings are consistent with the meta-analysis by Ntoumanis et al. (22), which found that attitudes, perceived norms, and self-efficacy to abstain from doping were significant predictors of doping intentions and behaviors. This further highlights the importance of understanding the discrepancy between *subjective* and *objective ADK*. Misaligned or inaccurate subjective knowledge, as evidenced in our study, could lead to flawed attitudes or distorted perceptions of control. These, in turn, may influence not only intentions but also actual doping behaviors, thereby increasing doping risk. The present study has identified specific areas of knowledge gaps that could serve as focal points for future educational interventions. Reconciling *subjective* and *objective ADK* is critical to both self-efficacies to avoid doping and to improve compliance, thereby reducing overall doping risk.

In recent years, WADA has emphasized the importance of starting education at an early age (20), underscoring the importance of early and sustained involvement in anti-doping efforts (4, 26, 53–56). A tiered approach targeting different stages of athlete development with corresponding educational goals has been recommended (57). One strategy might be to design and deliver these educational initiatives with *subjective ADK* in mind, potentially increasing their effectiveness and resonance among athletes. Looking further at demographic factors, it appears that athletes, particularly women, with 11–15 years of competitive experience, have a greater inclination to understand anti-doping rules and regulations. This tendency is consistent with a multi-country study showing that women and experienced athletes generally have more positive attitudes toward anti-doping education (31). Such findings are critical in tailoring and targeting anti-doping education initiatives for maximum effectiveness.

### 4.4. Future research directions

To better align *subjective* and *objective ADK* and further increase the effectiveness of anti-doping efforts, future research could delve deeper into understanding the long-term effects of different educational and moral interventions on knowledge retention and attitudes (26, 58).

### 4.5. Limitations of the study

Three limitations exist in this study. First, the ALPHA test designed to verify anti-doping knowledge after e-learning has a rather limited and straightforward question set. The item analysis indicated that some of the questions had a gentler difficulty level. This risks, among other things, that subjects will rate their level of knowledge higher than it actually is and thus underestimate the importance of the educational intervention.

Second, because *subjective ADKs* in this study are self-reported, the potential for bias should be considered. In particular, based on the *TPB*, this limitation of self-report should be considered as it may make it difficult to assess its impact on individual attitudes and behavioral intentions.

Third, the survey was conducted online, which, while convenient for data collection, may introduce bias. While the online format allows for easier distribution and quicker responses, it may also have made it easier for respondents to provide less thoughtful or even inauthentic responses. However, the online format did not significantly affect the quality of the data because the survey questions were similar in response format to established psychometric scales.

## 5. Conclusions

This study examined the relationship between *subjective ADK* and *objective ADK* among Japanese university athletes and revealed a discrepancy. Specifically, although athletes perceived themselves to have adequate AD knowledge, objective measures indicated otherwise. Such discrepancies, when considered in the context of the *TPB*, suggest potential shortcomings in the perceived behavioral control of athletes over their anti-doping knowledge. Consequently, even if athletes recognize doping as an egregious violation and possess attitudes and subjective norms consistent with anti-doping compliance, knowledge deficits may inadvertently skew behavioral intentions, potentially leading to unintentional ADRVs.

Understanding the nuances between *subjective* and *objective ADKs* for specific educational topics can provide a richer, more granular insight that enhances the effectiveness of anti-doping education programs. Based on the *TPB*, it is paramount to understand how *subjective ADV*, attitudes, social norms, and perceived behavioral control collectively shape anti-doping behavior. Designing educational programs that comprehensively address these elements is imperative to bridge the identified knowledge gap.

## Data Availability

The raw data supporting the conclusions of this article will be made available by the authors, without undue reservation.
