# Supplementary material for: The association between subjective anti-doping knowledge and objective knowledge among Japanese university athletes: a cross-sectional study
Source: Front Sports Act Living. 2023 Nov 16;5:1210390. doi: 10.3389/fspor.2023.1210390 (PMC10687363; doi:10.3389/fspor.2023.1210390)
Supplement: Supplementary file 1 [file Datasheet1.docx]

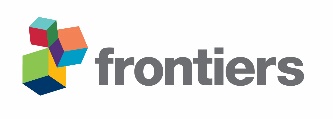
**Supplementary Table 1. Selection rate for each selected branch of the ALPHA question.**

| **No** | **ALPHA question content** | **n** | **(n%)** |
| --- | --- | --- | --- |
| **1** | **What is the philosophy behind anti-doping?** |  |  |
|  | a. To restrict the pharmaceutical industry’s access to athletes | 0 | 0% |
|  | b. To hold athletes to a higher standard than non-athletes | 2 | 0.41% |
|  | ***c. To protect the spirit of sport*** | 423 | ***87.04%*** |
|  | d. To promote discipline among athletes | 61 | 12.55% |
| **2** | **What is the purpose of the World Anti-Doping Code?** |  |  |
|  | a. To protect athletes’ fundamental right to participate in doping-free sports | 23 | 4.73% |
|  | b. To promote health and fairness and equality for athletes | 115 | 23.66% |
|  | c. To ensure harmonious and effective content anti-doping program at international level | 15 | 3.09% |
|  | ***d. All of the above*** | 333 | ***68.52%*** |
| **3** | **What is the Prohibited List?** |  |  |
|  | **a. The list of doctors who are not allowed to work with athletes because of doping sanctions** | 23 | 4.73% |
|  | b. The list of athletes that have been banned from competition | 29 | 5.97% |
|  | ***c. The list of substances and methods that are prohibited in competition and out of competition*** | 423 | ***87.04%*** |
|  | d. The list of support personnel who are not allowed to work with athletes because of doping sanctions | 11 | 2.26% |
| **4** | **What are the side effects of using anabolic steroids?** |  |  |
|  | a. Men with breasts and women with deep voices | 64 | 13.17% |
|  | b. Liver and heart failure | 110 | 22.63% |
|  | c. Violent mood swings | 91 | 18.72% |
|  | ***d. All of the above*** | 221 | ***45.47%*** |
| **5** | **What does TUE stand for?** |  |  |
|  | a. Team Update Exemption | 21 | 4.32% |
|  | b. Therapeutic Use Enhancement | 22 | 4.53% |
|  | ***c. Therapeutic Use Exemption*** | 433 | ***89.09%*** |
|  | d. Technical Use Exchange | 10 | 2.06% |
| **6** | **How can an athlete with a medical condition decide whether to take a medication?** |  |  |
|  | a. Athletes can take any medication for medicinal purposes | 16 | 3.29% |
|  | b. The medication is permitted if the medical condition would hinder performance in competition | 91 | 18.72% |
|  | ***c. The athlete should determine the need for the medication and seek a TUE*** | 345 | ***70.99%*** |
|  | d. The medication is permitted if it is prescribed by a doctor | 34 | 7.00% |
| **7** | **Who is responsible for the substances found in an athlete’s body?** |  |  |
|  | ***a. The athlete*** | 464 | ***95.47%*** |
|  | b. The doctor | 0 | 0% |
|  | c. The coach | 5 | 1.03% |
|  | d. The person who provided the substance | 17 | 3.50% |
| **8** | **What condition allows an athlete to refuse to be tested?** |  |  |
|  | a. Family commitments | 8 | 1.65% |
|  | b. Busy schedules | 16 | 3.29% |
|  | c. Academic obligations | 28 | 5.76% |
|  | ***d. Athletes cannot refuse testing*** | 434 | ***89.30%*** |
| **9** | **When must an athlete be notified of an upcoming test?** |  |  |
|  | a. 1 month prior | 67 | 13.79% |
|  | b. 7 days prior | 37 | 7.61% |
|  | c. 24 h prior | 48 | 9.88% |
|  | ***d. No advance notice is required*** | 334 | ***68.72%*** |
| **10** | **When do athletes have to tell their National Anti-Doping Organization where they will be living, training and competing?** |  |  |
|  | a. Athletes are not required to do this | 15 | 3.09% |
|  | ***b. When they are in a Registered Testing Pool (RTP)*** | 201 | ***41.36%*** |
|  | c. During any year when the Olympics are being held | 38 | 7.82% |
|  | d. All athletes must do this | 232 | 47.74% |
| **11** | **What are the athlete’s right when a positive test is returned?** |  |  |
|  | a. The right to have the B sample analyzed | 68 | 13.99% |
|  | b. The right to attend the opening and analysis of the B sample | 68 | 13.99% |
|  | c. The right to copies of the laboratory documentation package | 22 | 4.53% |
|  | ***d. All of the above*** | 328 | ***67.49%*** |
| **12** | **What is the requirement for laboratories that analyze blood or urine samples for doping control?** |  |  |
|  | a. The laboratory must be based in the country where the doping control took place | 45 | 9.26% |
|  | b. Any laboratory may analyze samples | 55 | 11.32% |
|  | c. The laboratory must be based in the athlete’s country | 28 | 5.76% |
|  | ***d. The laboratory must be accredited by WADA*** | 358 | ***73.66%*** |

*Note*.

The correct answer choices are marked in bold, underline, and italics.

n: number of eligible participants.

(n%): Percentage of eligible participants.

**Supplementary Table 2. Discrimination index of the ALPHA question.**

| **No** | **ALPHA question content** | **ALPHA**  **H*igh score group***  **(n = 182)** | **ALPHA**  ***Middle score group***  **(n = 91)** | **ALPHA**  ***Low score  group***  **(n = 213)** | **Index of  Discrimination** |
| --- | --- | --- | --- | --- | --- |
| **1** | **What is the philosophy behind anti-doping?** |  |  |  |  |
|  | a. To restrict the pharmaceutical industry’s access to athletes | 0 | 0 | 0.009 | -0.009 |
|  | b. To hold athletes to a higher standard than non-athletes | 0 | 0 | 1.099 | -1.099 |
|  | ***c. To protect the spirit of sport*** | ***0.906*** | ***0.877*** | ***0.821*** | ***0.084*** |
|  | d. To promote discipline among athletes | 0.094 | 0.165 | 0.170 | -0.075 |
| **2** | **What is the purpose of the World Anti-Doping Code?** |  |  |  |  |
|  | a. To protect athletes’ fundamental right to participate in doping-free sports | 0.038 | 0.030 | 0.098 | -0.060 |
|  | b. To promote health and fairness and equality for athletes | 0.113 | 0.194 | 0.455 | -0.342 |
|  | c. To ensure harmonious and effective content anti-doping program at international level | 0.019 | 0.030 | 0.045 | -0.026 |
|  | ***d. All of the above*** | ***0.830*** | ***0.746*** | ***0.402*** | ***0.428*** |
| **3** | **What is the Prohibited List?** |  |  |  |  |
|  | **a. The list of doctors who are not allowed to work with athletes because of doping sanctions** | 0.009 | 0.034 | 0.107 | -0.098 |
|  | b. The list of athletes that have been banned from competition | 0.057 | 0.045 | 0.098 | -0.042 |
|  | ***c. The list of substances and methods that are prohibited in competition and out of competition*** | ***0.915*** | 0.903 | ***0.759*** | ***0.156*** |
|  | d. The list of support personnel who are not allowed to work with athletes because of doping sanctions | 0.019 | ***0.019*** | 0.036 | -0.017 |
| **4** | **What are the side effects of using anabolic steroids?** |  |  |  |  |
|  | a. Men with breasts and women with deep voices | 0.104 | 0.142 | 0.134 | 0.070 |
|  | b. Liver and heart failure | 0.094 | 0.246 | 0.304 | 0.050 |
|  | c. Violent mood swings | 0.066 | 0.209 | 0.250 | -0.837 |
|  | ***d. All of the above*** | ***0.736*** | ***0.403*** | ***0.313*** | ***0.717*** |
| **5** | **What does TUE stand for?** |  |  |  |  |
|  | a. Team Update Exemption | 0.028 | 0.026 | 0.098 | -0.070 |
|  | b. Therapeutic Use Enhancement | 0.028 | 0.026 | 0.107 | -0.079 |
|  | ***c. Therapeutic Use Exemption*** | ***0.943*** | ***0.922*** | ***0.768*** | ***0.176*** |
|  | d. Technical Use Exchange | 0.000 | 0.026 | 0.027 | -0.027 |
| **6** | **How can an athlete with a medical condition decide whether to take a medication?** |  |  |  |  |
|  | a. Athletes can take any medication for medicinal purposes | 0.019 | 0.019 | 0.080 | -0.052 |
|  | b. The medication is permitted if the medical condition would hinder performance in competition | 0.028 | 0.224 | 0.250 | -0.222 |
|  | ***c. The athlete should determine the need for the medication and seek a TUE*** | ***0.906*** | ***0.716*** | ***0.509*** | ***0.397*** |
|  | d. The medication is permitted if it is prescribed by a doctor | 0.047 | 0.041 | 0.161 | -0.114 |
| **7** | **Who is responsible for the substances found in an athlete’s body?** |  |  |  |  |
|  | ***a. The athlete*** | ***0.981*** | 0.966 | ***0.902*** | ***0.079*** |
|  | b. The doctor | 0 | 0 | 0 | 0.000 |
|  | c. The coach | 0.009 | 0.011 | 0.009 | 0.001 |
|  | d. The person who provided the substance | 0.009 | 0.022 | 0.089 | -0.080 |
| **8** | **What condition allows an athlete to refuse to be tested?** |  |  |  |  |
|  | a. Family commitments | 0.009 | 0.011 | 0.036 | -0.026 |
|  | b. Busy schedules | 0.019 | 0.030 | 0.054 | -0.035 |
|  | c. Academic obligations | 0.028 | 0.045 | 0.116 | -0.088 |
|  | ***d. Athletes cannot refuse testing*** | ***0.943*** | ***0.914*** | ***0.795*** | ***0.149*** |
| **9** | **When must an athlete be notified of an upcoming test?** |  |  |  |  |
|  | a. 1 month prior | 0.085 | 0.131 | 0.205 | -0.120 |
|  | b. 7 days prior | 0.047 | 0.078 | 0.098 | -0.051 |
|  | c. 24 h prior | 0.057 | 0.101 | 0.134 | -0.077 |
|  | ***d. No advance notice is required*** | ***0.811*** | ***0.690*** | ***0.563*** | ***0.249*** |
| **10** | **When do athletes have to tell their National Anti-Doping Organization where they will be living, training and competing?** |  |  |  |  |
|  | a. Athletes are not required to do this | 0.019 | 0.026 | 0.054 | -0.035 |
|  | ***b. When they are in a Registered Testing Pool (RTP)*** | ***0.623*** | ***0.332*** | ***0.411*** | ***0.212*** |
|  | c. During any year when the Olympics are being held | 0.019 | 0.097 | 0.089 | -0.070 |
|  | d. All athletes must do this | 0.340 | 0.545 | 0.446 | -0.107 |
| **11** | **What are the athlete’s right when a positive test is returned?** |  |  |  |  |
|  | a. The right to have the B sample analyzed | 0.047 | 0.119 | 0.277 | -0.230 |
|  | b. The right to attend the opening and analysis of the B sample | 0.094 | 0.146 | 0.170 | -0.075 |
|  | c. The right to copies of the laboratory documentation package | 0.009 | 0.052 | 0.063 | -0.053 |
|  | ***d. All of the above*** | ***0.849*** | ***0.683*** | ***0.491*** | ***0.358*** |
| **12** | **What is the requirement for laboratories that analyze blood or urine samples for doping control?** |  |  |  |  |
|  | a. The laboratory must be based in the country where the doping control took place | 0.038 | 0.067 | 0.205 | -0.138 |
|  | b. Any laboratory may analyze samples | 0.047 | 0.116 | 0.170 | -0.054 |
|  | c. The laboratory must be based in the athlete’s country | 0.038 | 0.045 | 0.107 | -0.062 |
|  | ***d. The laboratory must be accredited by WADA*** | ***0.877*** | ***0.772*** | ***0.518*** | ***0.255*** |

*Note*.

The correct answer choices are marked in bold, underline, and italics.

The index of Discrimination was calculated based on (Kelly, 1939). Participants were grouped based on the ALPHA total score:

*High group*: Top 27% with scores less than 7 points.

*Middle group*: Middle 46%

*Low group*: Bottom 27% with scores of 11 points or more.

The discrimination index represents the difference in item correct answer rates between the *High group* and *Low group* was calculated.

Item difficulties are considered desirable and range between 0.2 and 0.8. The D index for No. 1, 3, 5, 7, and 8 was less than 0.2.

**Supplement Table 3.** **Mean and Cronbach’s alpha of the deleted items.**

| **ALPHA question item** | | **Reliability analysis** | | **Mean** | **SD** |
| --- | --- | --- | --- | --- | --- |
|  |  | **Corrected Item-Total Correlation** | **Cronbach’s alpha  if item deleted** |  |  |
| **1** | **What is the philosophy behind anti-doping?** | 0.147 | 0.607 | 0.870 | 0.336 |
| **2** | **What is the purpose of the World Anti-Doping Code?** | 0.339 | 0.571 | 0.690 | 0.465 |
| **3** | **What is the Prohibited List?** | 0.318 | 0.580 | 0.870 | 0.334 |
| **4** | **What are the side effects of using anabolic steroids?** | 0.230 | 0.597 | 0.450 | 0.498 |
| **5** | **What does TUE stand for?** | 0.324 | 0.580 | 0.890 | 0.312 |
| **6** | **How can an athlete with a medical condition decide whether to take a medication?** | 0.280 | 0.584 | 0.710 | 0.454 |
| **7** | **Who is responsible for the substances found in an athlete’s body?** | 0.281 | 0.593 | 0.950 | 0.208 |
| **8** | **What condition allows an athlete to refuse to be tested?** | 0.295 | 0.585 | 0.890 | 0.309 |
| **9** | **When must an athlete be notified of an upcoming test?** | 0.229 | 0.596 | 0.690 | 0.464 |
| **10** | **When do athletes have to tell their National Anti-Doping Organization where they will be living, training and competing?** | 0.105 | 0.626 | 0.410 | 0.493 |
| **11** | **What are the athlete’s right when a positive test is returned?** | 0.303 | 0.579 | 0.670 | 0.469 |
| **12** | **What is the requirement for laboratories that analyze blood or urine samples for doping control?** | 0.410 | 0.555 | 0.740 | 0.441 |

*Note*. SD: standard deviation.

Cronbach’s alpha coefficient for the 12 ALPHA items was 0.609. I-T correlation analysis showed that question No. 12 had a rather strong positive correlation with the total score. No2-9 and 11 showed weak positive correlations. No 1 and 10 showed no correlation.

**Supplement Table 4. Population distribution by participating athletic event.**

| **Athletic event** | **n** | **(n%)** |
| --- | --- | --- |
| Athletics | 111 | 22.84% |
| Football | 73 | 15.02% |
| Basketball | 42 | 8.64% |
| Baseball | 42 | 8.64% |
| Volleyball | 41 | 8.44% |
| Swimming | 26 | 5.35% |
| Handball | 18 | 3.70% |
| Kendo | 17 | 3.50% |
| Gymnastics | 17 | 3.50% |
| Tennis | 15 | 3.09% |
| Judo | 13 | 2.67% |
| Soft tennis | 11 | 2.26% |
| Badminton | 9 | 1.85% |
| Table Tennis | 6 | 1.23% |
| Futsal | 5 | 1.03% |
| Rhythmic gymnastics | 5 | 1.03% |
| Softball | 4 | 0.82% |
| Rugby football | 3 | 0.62% |
| Dance sports | 2 | 0.41% |
| Karate | 2 | 0.41% |
| Skiing | 2 | 0.41% |
| Water polo | 2 | 0.41% |
| American football | 1 | 0.21% |
| Bicycling | 1 | 0.21% |
| Cheer dance | 1 | 0.21% |
| Cheerleading | 1 | 0.21% |
| Dodgeball | 1 | 0.21% |
| Gate ball | 1 | 0.21% |
| Ice hockey | 1 | 0.21% |
| Rowing | 1 | 0.21% |
| Sport climbing | 1 | 0.21% |
| Wushu Tai Chi | 1 | 0.21% |
| Other sports | 10 | 2.06% |
